# Profil des diabétiques en hémodialyse chronique: étude multicentrique au Maroc

**DOI:** 10.11604/pamj.2014.17.125.3792

**Published:** 2014-02-21

**Authors:** Nadia Kabbali, Souad Mikou, Nada Tazi El Pardiya, Ghita El Bardai, Mohamed Arrayhani, Tarik Sqalli Houssaini

**Affiliations:** 1 Service de Néphrologie-Dialyse-Transplantation du CHU Hasan II de Fès, Fès, Maroc; 2 Laboratoire d'Epidémiologie de la Faculté de Médecine et de Pharmacie de Fès, Fès, Maroc

**Keywords:** hémodialyse, diabète, insuffisance rénale chronique, prévalence, comorbidités cardiovasculaires, hemodialysis, diabetes, chronic renal failure, prevalence, cardiovascular comorbidity

## Abstract

**Introduction:**

Le diabète représente la première cause de mortalité par insuffisance rénale au Maroc. Sa prévalence selon l'Enquête Nationale sur la Population et la Santé Familiale de 2011 est de 3,3% [1]. Le but de ce travail est de déterminer la prévalence et d’étudier le profil clinique des diabétiques en hémodialyse chronique au Maroc.

**Méthodes:**

Il s'agit d'une étude transversale multicentrique incluant 2066 hémodialysés chroniques dans les 39 centres d'hémodialyse de quatre régions marocaines.

**Résultats:**

La prévalence du diabète en hémodialyse est 21,6%. L’âge moyen 59±13,2 ans (26-87). Le sex-ratio est de 1,9. L'IMC moyen est de 24,5 ± 4,4 kg/m^2^ (15-41). 42 patients sont porteurs d′une hépatite virale. La durée moyenne en HD est 39,3± 67 mois. 57% des patients gardent une diurèse résiduelle versus 43% chez les non diabétiques. Par rapport à ces derniers, nous avons noté plus d'HTA (64% versus 45%), plus de complications cardiovasculaires (23% versus 12%), un âge plus avancé à l'initiation de l'hémodialyse (55,5 versus 47 ans) et un taux de FAV proximales plus important.

**Conclusion:**

La prévalence des diabétiques en hémodialyse est relativement élevée au Maroc sans tenir compte des patients qui ne bénéficient pas d’épuration extra-rénale pour des raisons socio-économiques. Le taux élevé de mortalité est imputable au retard et/ou à l'absence de la prise en charge néphrologique des diabétiques. Dans nos régions où le système sanitaire dispose de faible moyen, l'accent doit être mis sur le dépistage précoce de la néphropathie chez le diabétique.

## Introduction

Le diabète constitue la première cause d'insuffisance rénale chronique (IRC) dans les pays industrialisés [2]. Au Maroc, sa prévalence selon l'Enquête Nationale sur la Population et la Santé Familiale (ENPSF-2011) est de 3,3% [1]. L′incidence de la néphropathie diabétique au stade terminal est variable selon les pays, incidence qui a tendance à augmenter en rapport avec l'amélioration de la survie des patients diabétiques. Cependant, la survie et la qualité de vie de ces patients en hémodialyse restent significativement moins bonnes que celles des patients non diabétiques. Les complications cardiovasculaires associées, les problèmes liés aux abords vasculaires, la prise de poids interdialytique et les épisodes fréquents d'hypotension intradialytique expliquent ce pronostic défavorable. Le but de ce travail est de déterminer la prévalence et d’étudier le profil clinique des diabétiques en hémodialyse chronique au Maroc.

## Méthodes

Il s'agit d'une étude transversale multicentrique incluant tous les patients hémodialysés chroniques dans les 39 centres d'hémodialyse de quatre régions parmi les 16 régions administratives marocaines: Fès - Boulemane, Meknès - Tafilalt, Taza - Taounat - Al Hoceima et la région de l'Oriental. La population totale de ces quatre régions selon le recensement général de la population au Maroc est de 7 405 410 habitants soit 29,5% de la population marocaine [3]. Un consentement oral éclairé des patients a été obtenu. Nous avons collecté les données sociodémographiques, les comorbidités ainsi que les données liées à l'hémodialyse. Ensuite, nous avons comparés les caractéristiques des patients diabétiques à celles des patients non diabétiques. L'analyse statistique a été réalisée par le logiciel SPSS version 19. Nous avons utilisé le test de Student pour les variables quantitatives et le test de Chi^2^ pour les variables qualitatives. Le seuil de significativité est considéré positif si le p est inférieur à 0,05.

## Résultats

**Figure 1:**
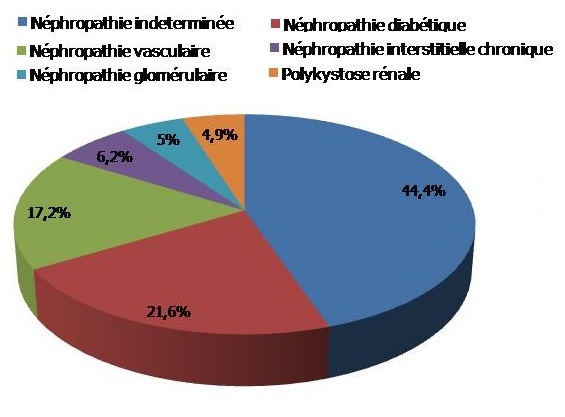
Néphropathie initiale chez nos patients hémodialysés

Nous avons inclus 2066 hémodialysés chronique dont 447 sont diabétiques, soit une prévalence de 21,6%. Les autres causes d'IRCT sont schématisées sur la Figure 1. L’âge moyen des diabétiques est de 59 ± 13,2 ans avec des extrêmes de 26 à 87 ans. Ils ont débuté l'hémodialyse à l’âge de 55,5 ans en moyenne. Le sex-ratio est de 1,9 soit 291 hommes (65,1%) et 156 femmes (34,9%). Il s'agit d'un diabète de type 2 dans les trois quarts des cas, et les patients bénéficient d'une couverture sociale dans la moitié des cas. L'IMC moyen de nos patients est de 24,5 ± 4,4 kg/m^2^. 21% sont obèses et 78% en surpoids. Nous avons noté un IMC < 18 chez 16 patients, tous étaient âgés de plus de 80 ans. Les autres facteurs de risque cardiovasculaires étaient l'HTA dans 64% des cas, le tabac dans 16,3% des cas et la consommation d'alcool dans 6% des cas. 22,6% des patients présentent une comorbidité cardiovasculaire. Il s'agit d'une cardiopathie ischémique dans 62,4% des cas et d'une insuffisance cardiaque dans 27,7%. 42 patients (9,4%) sont porteurs d′une hépatite virale (9 HBV, 33 HCV dont une co-infection).

La durée moyenne en hémodialyse est de 39,3± 67 mois. 57% des patients gardent une diurèse résiduelle. Le nombre de séances hebdomadaires est de trois dans 260 cas (58,2%). 12 patients sont dialysés sur cathéter permanent. Les autres patients sont dialysés sur une fistule artério-veineuse (FAV) qui est distale dans 74,8%, et proximale dans 22,5% dont cinq étaient prothétiques. Dans 76,2%, il s′agit d'une première FAV, et dans 17,2% d'une deuxième FAV. C’était une troisième FAV chez 14 patients, et d'une quatrième chez 12 patients. Chez deux patients, il s'agissait de la cinquième ou de la sixième FAV. 317 patients (71%) disent souhaiter une transplantation rénale. Cependant, seuls 107 (23,9%) affirment que le sujet a été abordé par leur médecin, alors que dix patients pensent que le diabète est une contre indication à la transplantation. 57% des patients gardent une diurèse résiduelle versus 43% chez les non diabétiques. L'analyse univariée a également montré que, par rapport aux patients non diabétiques, les patients diabétiques présentaient plus d'HTA, plus de comorbidités cardiovasculaires, un âge plus avancé à l'initiation de l'HD, un IMC plus élevé et un taux de FAV proximales plus important (Tableau 1). En analyse multivariée, la diurèse résiduelle et le siège de la FAV n’étaient plus significatifs.

**Tableau 1:** Caractéristiques des hémodialysés diabétiques versus les non diabétiques en analyse univariée

	Diabétiques (n = 447)	Non diabétiques (n = 1618)	p
Diurèse résiduelle	57%	42,9%	<0,001
HTA	64%	45,4%	<0,001
Complication cardiovasculaire	22,6%	12,3%	<0,001
Nombre de FAV ≥ 3	6,9%	6,8%	0,07
FAV proximale	19,9%	16,7%	0,05
Age à l'initiation de l'hémodialyse (ans)	55,5	47	<0,001
IMC (Kg/m^2^)	24,5	22,4	<0,001

## Discussion

La prévalence des diabétiques en IRCT est relativement élevée au Maroc sans tenir compte des patients qui ne bénéficient pas d’épuration extra-rénale pour des raisons socio-économiques. Cette prévalence est variable selon les pays: 44% aux Etats-Unis en 2008 [4], 34% en Australie-Nouvelle-Zélande en 2008 [5], 11,8% à 35,5% selon les pays en Europe en 2007 [6], et 35% en France en 2006 [7]. Cette prévalence a tendance à augmenter au fil des années. En effet, aux USA, le pourcentage de patients hémodialysés diabétiques augmente de plus de 1% par an [2]. En Europe, cette prévalence a doublé au cours de ces dix dernières années [8]. Ce phénomène peut être expliqué par plusieurs facteurs: l'augmentation de la prévalence du diabète de type 2 dans la population générale, l'amélioration de la survie des patients diabétiques par un meilleur contrôle de l'HTA et des complications cardiovasculaires, ce qui fait qu'ils vivent assez longtemps pour commencer la dialyse, et enfin l'amélioration de l'accessibilité au traitement par hémodialyse. Concernant la variabilité de prévalence notée entre les différents pays, elle peut être expliquée par l'absence d'homogénéité de codage de la néphropathie initiale en vue de comparaisons internationales.

En effet, dans l’étude française de Couchoud [7], la néphropathie initiale n'a été codée comme d'origine diabétique que chez 66% des personnes diabétiques de type 1 et 53% des diabétiques de type 2. Ces chiffres sont inférieurs à ceux retrouvés dans le registre Anzdata d'Australie et Nouvelle- Zélande, où 94% des personnes diabétiques de type 1 et 74% des diabétiques de type 2 ont une néphropathie initiale déclarée comme d'origine diabétique [5]. Or ces diagnostics de néphropathie sont en majorité des diagnostics de présomption, puisque seuls 9% des personnes diabétiques en France et 16% en Australie et Nouvelle-Zélande ont été biopsiées. Il convient de ce fait d'interpréter avec prudence la distribution des néphropathies initiales dont le codage peut varier selon les pratiques médicales en l'absence de définition standardisée sur le codage des maladies. L'utilisation de la néphropathie initiale quantifie uniquement le diabète comme cause initiale de la maladie rénale chronique. Mais un diabète associé mal équilibré constitue un facteur de risque de progression de toute néphropathie sous-jacente jugée comme cause initiale de l'IRCT [9].

Dans notre étude, comparés aux patients en IRCT sans diabète, les patients diabétiques étaient plus âgés à l'initiation de l'hémodialyse, plus souvent en surcharge pondérale, et avaient significativement plus de comorbidités cardiovasculaires. L′âge plus élevé des malades à l'initiation de l'hémodialyse a été rapporté par plusieurs autres auteurs. Il peut être expliqué par la référence tardive des malades en néphrologie, et l′initiation de l′hémodialyse au stade de complications le plus souvent. Dans une autre série marocaine [10], seulement 32% des patients avaient un suivi spécialisé d′au moins quatre mois avant la mise en hémodialyse. Ces résultats sont comparables à ceux d'autres séries européennes et américaines [11–14]. Ils témoignent d′une situation fréquente et internationalement constatée. Concernant les comorbidités cardiovasculaires, comme dans notre série, plusieurs études ont noté un taux plus élevé chez les personnes diabétiques [7, 15]. Ces conditions expliquent en grande partie le pronostic sévère de ces personnes en dialyse et leur accès moins aisé à la transplantation [16]. Etant donné l'augmentation actuelle de la prévalence du diabète, la fréquence de ces comorbidités va également augmenter. Des interventions de prévention secondaire et tertiaire doivent être privilégiées dans cette population.

D'autre part, le diabète apparait comme un facteur altérant la survie des FAV. La médiacalcose, la dysfonction endothéliale, ainsi que l′augmentation du stress oxydatif responsable d′une augmentation des événements thrombotiques, contribuent à l′échec des abords vasculaires chez les diabétiques [17–19]. Dans notre série, ceci a été reflété par la difficulté de création d'une FAV distale chez les patients diabétiques par rapport aux non diabétiques. En effet, le taux de FAV proximales était plus élevé chez les patients diabétiques. Une exploration systématique du membre supérieur avant la confection de l′abord vasculaire diminuerait de façon significative le risque d′échec de 23% selon Branger [20].

## Conclusion

La prévalence des diabétiques en hémodialyse est relativement élevée au Maroc, et elle devrait continuer à croître dans les années à venir. Le taux élevé de mortalité est imputable au retard et/ou à l'absence de la prise en charge néphrologique des diabétiques. Cette étude confirme également l'impact péjoratif du diabète associé à une maladie rénale chronique. Elle souligne l'importance d'organiser une prise en charge adaptée en amont afin de prévenir l'apparition des comorbidités cardiovasculaires et de préparer au mieux le patient au traitement de suppléance.
